# Association of genetic polymorphisms of *eNOS* with glaucoma

**Published:** 2011-01-13

**Authors:** Qiong Liao, Dai-Hong Wang, Han-Jun Sun

**Affiliations:** 1 Department of Ophthalmology, The Affiliated Xinqiao Hospital of the 3rd Military Medical College, Chongqi, P.R. China; 2 Department of Urology, The Affiliated Xinqiao Hospital of the 3rd Military Medical College, Chongqi, P.R. China

## Abstract

**Purpose:**

Several studies suggest that vascular dysregulation play a role in the etiology of glaucoma. In the present study, we aimed to investigate the association of endothelial nitric oxide synthase (*eNOS*) gene polymorphisms with primary open angle glaucoma (POAG) and primary closed angle glaucoma (PCAG).

**Methods:**

There were 102 POAG and 88 PCAG patients, diagnosed on the basis of clinical history, raised intraocular pressure (IOP), cup-to-disc ratio (CDR) and visual field defects, and 120 age- and sex-matched control subjects genotyped for 5 tagging single nucleotide polymorphisms (SNPs; rs743507, rs3793342, rs11771443, rs7830, and rs3918188) of the human *eNOS* gene.

**Results:**

rs3793342, rs743507, rs11771443, rs7830, and rs3918188 were not found to be associated with POAG or with PCAG. In the haplotype-based case-control analysis, the frequency of the C-T haplotype established by rs3793342 and rs11771443 was significantly higher for POAG patients than for control subjects (p<0.001, OR=5.111, 95%CI=1.766~14.788).

**Conclusions:**

The C-T haplotype established by rs3793342 and rs11771443 may be genetic marks of POAG in the Han Chinese population.

## Introduction

Glaucoma, an irreversible retinal deterioration which results in progressive visual field loss along with decreased contrast and color sensitivity, is a multifactorial optic neuropathy characterized by apoptotic cell death of the retinal ganglion cells (RGCs) in the optic disc or retinal nerve fiber [1,2]. Previous studies suggested that vascular dysregulation play an important role in the etiology of glaucoma [3,4]. Recently, activation of nitric oxide synthase (NOS), an enzyme associated with the death of RGC caused by ischemic injury, has been reported as one potential mechanism of glaucomatous damage to the retina and optic nerve. Several isoforms of NOS have been reported in abundance in almost all layers of the retina, but the circulating NO is synthesized solely in the vascular endothelium through the action of the endothelial nitric oxide synthase (eNOS) on the substrate L-arginine [5]. Previous studies have revealed that polymorphisms in the *eNOS* gene may alter *eNOS* expression and thus cause a decrease in NO synthesis [3], which may related to cardiovascular disease. Nevertheless, an abundance of NOS also had been found in the optic nerve head vessels of primary glaucoma patients, supporting the idea that the optic nerve damage in glaucoma can be related to *eNOS* overexpression [6].

Recently, Ayub et al. [7] reported an association of an *eNOS* genetic polymorphism with glaucoma in Pakistani cohorts. But the relationship between *eNOS* polymorphisms and glaucoma in Han Chinese is unclear. The present study was conducted to determine whether *eNOS* polymorphisms were associated with POAG and PCAG in the Han Chinese population.

## Methods

### Subjects

The study followed the tenets of the Declaration of Helsinki with written informed consent obtained from all patients or from their parents if their age was less than 18. The patients and controls were randomly selected from the Affiliated Xinqiao Hospital of the 3rd Military Medical College. The participants included 120 unaffected controls, 102 POAG patients, and 88 PCAG patients. The patients were selected on the basis of their clinical history, cup-to-disc ratio (CDR) evaluation, visual field evaluation, and elevated IOP, and categorized into POAG and PCAG groups based on gonioscopic findings. In addition, to rule out any ocular anomaly, the controls also underwent applanation tonometery, slit lamp examination, CDR measurement and visual field assessment.

### Genotyping

**Figure 1:**
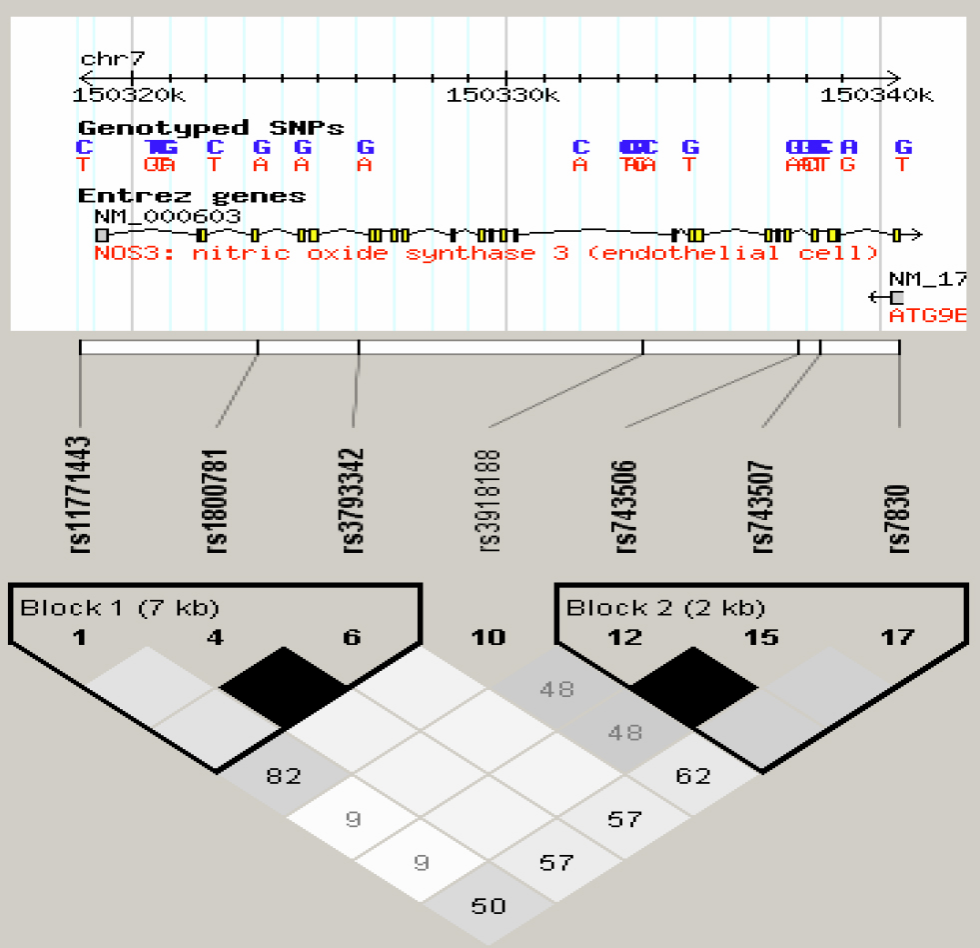
Genetic variation in the human *eNOS* gene. LD blocks across the locus in Chinese Han. LD blocks derived by solid spline method in Haploview. LD value shown: r^2^*100; r^2^ color scheme: r^2^=0: white; 0<r^2^<1: shades of gray; r^2^=1: black.

There are 253 single nucleotide polymorphisms (SNPs) for human *eNOS* listed in the National Center for Biotechnology Information SNP database. It has been observed that adjacent SNPs are often highly correlated. To reduce genotyping cost, many algorithms have been developed to select a smallest set of SNPs such that all the other SNPs can be inferred from them. The selected SNPs are called tag SNPs. As described on the website a tag SNP is a representative SNP in a region of the genome with high linkage disequilibrium (the non-random association of alleles at two or more loci). It is possible to identify genetic variation without genotyping every SNP in a chromosomal region. Tag SNPs are useful in whole-genome SNP association studies in which hundreds of thousands of SNPs across the entire genome are genotyped. For this reason, the International HapMap Project hopes to use tag SNPs to discover genes responsible for certain disorders. We selected the tag SNPs of *eNOS* on the International HapMap Project website using phase III database and analyzed these SNPs with Haploview 4.2 software. We obtained five tagging SNPs (rs743507, rs3793342, rs11771443, rs7830, and rs3918188) for Han Chinese using minor allele frequency (MAF) ≥0.05 and linkage disequilibrium patterns with *r*^2^≥0.8 as a cutoff. As shown in Figure 1, these five SNPs were located in two haplotype-blocks.

Genomic DNA was extracted from the peripheral blood leukocytes using a DNA extraction Kit (Beijing Bioteke Co. Ltd., Beijing, China). Quantification of extracted DNA was performed using a NanoDrop ND-1000 spectrophotometer (NanoDrop Technologies, Wilmington, DE). Genotyping was performed using the TaqMan® SNP Genotyping Assay (Applied Biosystems Inc., Foster City, CA) as described previously [8,9]. Briefly, The TaqMan SNP Genotyping Assays were performed using the method of Taq amplification. In the 5’nuclease assay, discrimination occurs during the polymerase chain reaction (PCR) because of allele-specific fluorogenic probes that, when hybridized to the template, are cleaved by the 5' nuclease activity of Taq polymerase. The probes contain a 3' minor groove-binding group (MGB) that hybridizes to single-stranded targets with greater sequence-specificity than ordinary DNA probes. This reduces nonspecific probe hybridization, and results in low background fluorescence in the 5' nuclease PCR assay. Cleavage results in increased emission of a reporter dye. Each 5' nuclease assay requires 2 unlabeled PCR primers and 2 allele-specific probes. Each probe is labeled with 2 reporter dyes at the 5' end.  In the present study, VIC and FAM were used as the reporter dyes. The primers and probes used in the TaqMan® SNP Genotyping Assays (ABI) were chosen based on information available at the ABI website. PCR amplification was performed using 6 µl of TaqMan® Universal Master Mix, No AmpErase® UNG (2×; ABI) in a 12 µl final reaction volume containing 2 ng of DNA, 0.22 µl of TaqMan® SNP Genotyping Assay Mix (20× or 40×), primers at a concentration of 900 nmol/l each, and probes at a final concentration of 200 nmol/l each. Thermal cycling conditions were as follows: 50 °C for 2 min; 95 °C for 10 min; 40 cycles of 95 °C for 15 s; and 62 °C for 1 min. Thermal cycling was performed using the GeneAmp 9700™ system.

### Statistical analysis

Hardy–Weinberg equilibrium (HWE) was tested for each polymorphism by the χ^2^ test. Allele or genotype frequencies between patients and controls were compared by the χ^2^ test or Fisher’s exact test. SPSS version 15.0 software (SPSS Inc., Chicago, IL) was used. Pairwise linkage disequilibrium (LD) estimation and expectation-maximization (EM)-based haplotype association analysis were performed using Haploview 4.2 [10] and SHEsis software [11,12]. In the haplotype-based case control analysis, haplotypes with a frequency of <0.03 were excluded. The frequency distribution of the haplotypes was calculated by performing a permutation test using the bootstrap method. In addition, logistic regression analysis was performed to calculate the OR value and its 95% confidence interval. To analysis the association of these five SNPs with POAG and PCAG, three models were presented such as the general model (common allele homozygotes coded as 1, heterozygotes as 2, and recessive allele homozygotes as 3), the dominant (common allele homozygotes coded as 1 and heterozygotes and recessive allele homozygotesas 2), and the recessive model (common allele homozygotes and heterozygotes coded as 1 and recessive allele homozygotes as 2). To assess the association of each SNP with POAG and PCAG, we used a Bonferroni correction to control for the number of variants tested; this was 5, so the probability value, 0.01, was considered to be significant.

## Results

The characteristics of the 310 study participants were shown in Table 1. Patients with POAG and PCAG and the controls were genotyped for five tagging SNPs of *eNOS*. Neither the genotype nor the allele frequencies of these 5 SNPs was significantly different between the control and the POAG patients (all p>0.01). There were not significant difference between the control subjects and the POAG/PCAG patients not only in the dominate model but also in the recessive model (all p>0.01; Table 2).

**Table 1:** Characteristics of study participants.

**Group**	**POAG**	**PCAG**	**Controls**
Number of subjects	102	88	120
Age at diagnosis (years)	59.1±17.3	60.7±14.7	65.6±16.4
Sex (M/F)	82/20	70/18	91/29
Highest IOP (mmHg)	24.3±8.2	28.5±8.8	15.6±3.2
Vertical cup-disc ratio	0.81±0.10	0.85±0.09	0.41±0.07

Note: 1 mmHg=0.133 kPa; For controls, age at diagnosis refers to age at inclusion.

**Table 2:** Frequencies of genotypes and alleles of the *eNOS* gene.

**Polymorphisms**	**Control (n=120)**	**POAG (n=102)**	**p# (χ2)**	**OR (95% CI)**	**PCAG (n=88)**	**p (χ2)**		**OR (95% CI)**
rs743507								
**Genotypes**								
AA	81 (0.675)	70 (0.686)	0.263 (2.675)	0.205 (0.023–1.872) p=0.122*	62 (0.705)	0.570 (1.223)		0.361 (0.032–4.405) p=0.390*
AG	38 (0.317)	28 (0.275)			24 (0.273)			
GG	1 (0.008)	4 (0.039)		1.053 (0.598–1.856) p=0.858**	2 (0.023)			1.148 (0.632–2.084) p=0.650**
**Alleles**								
A	0.833	0.822	0.785 (0.074)	0.933 (0.569–1.531)	0.841	0.836 (0.043)		1.057 (0.624–1.792)
G	0.167	0.178			0.159			
rs3793342								
**Genotypes**								
CC	91 (0.758)	66 (0.647)	0.045 (6.203)	7.435 (0.880–62.839) p=0.032*	60 (0.682)	0.388 (1.893)		0.361 (0.032–4.405) p=0.390*
CT	28 (0.233)	30 (0.294)			26 (0.295)			
TT	1 (0.008)	6 (0.059)		1.712 (0.956–3.067) p=0.069**	2 (0.023)			1.494 (0.370–1.261) p=0.222**
**Alleles**								
C	0.875	0.794	0.021 (5.310)	1.814 (1.088–3.030)	0.830	0.192 (0.695)		0.695 (0.401–1.203)
T	0.125	0.206			0.170			
rs11771443								
**Genotypes**								
CC	30 (0.250)	27 (0.265)	0.952 (0.085)	0.982(0.531–1.816) p=0.953*	23 (0.261)	0.965 (0.071)		1.084 (0.565–2.077) p=0.809*
CT	61 (0.508)	50 (0.490)			45 (0.511)			
TT	29 (0.242)	25 (0.245)		1.080 (0.591–1.975) p=0.802**	20 (0.227)			1.062 (0.565–1.993) p=0.853**
**Alleles**								
C	0.504	0.510	0.906 (0.014)	1.023 (0.704–1.486)	0.517	0.795 (0.067)		1.053 (0.713–1.554)
T	0.496	0.490			0.483			
rs7830								
**Genotypes**								
AA	23 (0.192)	18 (0.176)	0.922 (0.163)	0.901(0.507–1.560) p=0.721*	16 (0.182)	0.967 (0.068)		0.930 (0.510–1.695) p=0.813*
AC	62 (0.517)	52 (0.510)			45 (0.511)			
CC	35 (0.292)	32 (0.314)		0.904 (0.457–1.788) p=0.771**	27 (0.307)			0.937 (0.462–1.900) p=0.857**
**Alleles**								
A	0.450	0.431	0.694 (0.155)	0.927 (0.634–1.135)	0.438	0.780 (0.064)		0.951 (0.643–1.406)
C	0.550	0.569			0.562			
rs3918188								
**Genotypes**								
AA	5 (0.042)	4 (0.039)	0.965 (0.071)	1.065 (0.278–4.076) p=0.926*	4 (0.045)	0.942 (0.119)		0.913 (0.238–3.503) p=0.894*
AC	45 (0.375)	40 (0.392)			31 (0.352)			
CC	70 (0.583)	58 (0.569)		0.941 (0.552–1.606) p=0.825**	53 (0.602)			1.082 (0.618–1.894) p=0.784**
**Alleles**								
A	0.229	0.235	0.879 (0.023)	1.035 (0.665–1.610)	0.222	0.855 (0.033)		0.958 (0.601–1.526)
C	0.771	0.765			0.778			

*p: indicated the P value in dominate model; **p: indicated the p value in recessive model, ^#^p: indicated the p value in general model.

In the haplotype-based case-control analysis, for these five tagging SNPs located on two haplotype blocks, respectively, rs3793342 and rs11771443 located in block one and rs743507 and rs7830 located block two, but rs3918188 was not located in these two blocks (Figure 1). Haplotypes were established through the use of 4 SNPs (rs3793342 and rs11771443 in the block one; rs743507 and rs7830 in the block two; Table 3). The frequency of the C-T haplotype established by rs3793342 and rs11771443 was significantly higher for POAG patients than for control subjects (p<0.001, OR=5.111, 95%CI=1.766~14.788). However, the frequency of other haplotypes were not significantly different between the control subjects and the POAG/PCAG patients (all p>0.01).

**Table 3:** Haplotype distribution of the control and POAG and PCAG patients.

	**Frequency**				**Frequency**		
**Haplotypes**	**control**	**POAG**	**p**	**OR [95% CI]**	**PCAG**	**p**	**OR (95% CI)**
**Block 1 (rs3793342-rs11771443)**							
C-C	0.486	0.423	0.184	0.775 [0.532~1.129]	0.484	0.970	0.993 [0.673~1.465]
C-T	0.018	0.087	<0.001	5.111 [1.766~14.788]	0.033	0.337	1.834 [0.523~6.434]
T-C	0.389	0.371	0.699	0.927 [0.631~1.362]	0.346	0.363	0.829 [0.553~1.242]
T-T	0.107	0.119	0.683	1.130 [0.627~2.038]	0.137	0.341	1.334 [0.737~2.414]
**Block 2 (rs743507- rs7830)**							
A-A	0.292	0.292	0.999	1.000 [0.663~1.507]	0.302	0.756	1.070 [0.698~1.640]
A-C	0.541	0.531	0.837	0.962 [0.661~1.398]	0.539	0.904	1.025 [0.691~1.520]
G-A	0.158	0.139	0.584	0.863 [0.510~1.462]	0.136	0.571	0.852 [0.489~1.484]
G-C	0.009	0.037	0.041	4.331 [0.935~20.072]	0.023	-	-

### Limitations of this study

The present study was limited by the relatively small sample size. This may have led to weak statistical significance and wide CIs when estimating odds ratios.

## Discussion

The main findings in the present study were the C-T haplotype established by rs3793342 and rs11771443 may be genetic marks of POAG in the Han Chinese population.

The foundation for human studies examining putative causative genes that may be involved in glaucoma is based on a candidate gene approach. This involves selecting a functionally relevant gene to study and subsequently investigating its association with the glaucoma. The genes for *eNOS* present candidates for glaucoma because it is the gene encoding one important factor-eNOS, which is involved in different processes, like neurotransmission, the regulation of vascular tone, vasodilatation and apoptosis. In addition, it also regulates blood flow to the ocular tissues and has been implicated in the pathogenesis of cardiovascular diseases and different neurodegenerative disorders, such as diabetic retinopathy [13], glaucoma [14], and migraines [15]. And its downstream product-Nitric Oxide (NO) is responsible for maintaining arteries’ vasodilation, which keeps the ocular blood flow constant. When the endothelial function is deregulated, the blood supply to the tissue is altered and impaired blood flow damages the optic nerve and leads to the development of glaucomatous changes in the optic nerve, which then results in an increase in the CDR. Liu et al. [16] demonstrated that RGC degeneration in the glaucomatous optic nerve head of POAG patients clearly corresponds to excess plasma NO-mediated neurotoxicity. However, the authors did not conduct any genetic studies to find the susceptible loci.

Plasma NO levels are regulated by eNOS, therefore genetic polymorphisms of *eNOS* that enhances *eNOS* expression would contribute to NO mediated toxicity. The previously study [17] indicated that a 27-bp variable number of tandem repeat (VNTR) polymorphism in intron 4 of *eNOS* significantly influences plasma NO levels. Furthermore, Ayub and his colleagues [7] also found this VNTR polymorphism was associated with both POAG and PCAG in Pakistani cohorts. And Sakai et al. [18] found the T-786C polymorphism of *eNOS* to be a risk factor of non-arteritic anterior ischemic optic neuropathy (NAION) disease. However, Logan et al. [19] were unable to show a significant association between *eNOS* polymorphism T-786C and VNTR repeat polymorphism and glaucoma. Similarly, Sena et al. [20] and Lin et al. [21] did not find any association between POAG and *eNOS* intron 4 VNTR. Nevertheless, their studies were based on single SNP analysis rather than haplotypes approach. Morris et al. [22] reported that for genes with multiple susceptibilities, analysis based on haplotypes has advantages over analysis based on individual SNP.

In the present study, we genotyped 5 SNPs in Han Chinese subjects, and assessed the association between *eNOS* and glaucoma using a haplotype-based case-control analysis based on the tagging SNP approach. We found the the frequency of the C-T haplotype established by rs3793342 and rs11771443 was significantly higher for POAG patients than for control subjects. But we did not find any association between PCAG and *eNOS*. This result was not in line with Ayub et al. [7] who found an association of *eNOS* with not only POAG but also PCAG. The reason for this discrepancy is currently unclear, but may be due to, at least in part, the different genetic background of ethnicities under study.

### Conclusion

Our study indicated that the C-T haplotype established by rs3793342 and rs11771443 may be a genetic mark of POAG in the Han Chinese population.
